# Trichlorido(4,4′-dimethyl-2,2′-bipyridine-κ^2^ *N*,*N*′)(dimethyl sulfoxide-κ*O*)indium(III)

**DOI:** 10.1107/S1600536808029553

**Published:** 2008-09-20

**Authors:** Roya Ahmadi, Khadijeh Kalateh, Anita Abedi, Vahid Amani, Hamid Reza Khavasi

**Affiliations:** a Islamic Azad University, Shahr-e-Rey Branch, Tehran, Iran; b Department of Chemistry, Islamic Azad University, North Tehran Branch, Tehran, Iran; c Department of Chemistry, Shahid Beheshti University, Tehran 1983963113, Iran

## Abstract

In the mol­ecule of the title compound, [InCl_3_(C_12_H_12_N_2_)(C_2_H_6_OS)], the In^III^ atom is six-coordinated in a distorted octa­hedral configuration by two N atoms from the chelating 4,4′-dimethyl-2,2′-bipyridine ligand, one O atom from dimethyl sulfoxide and three Cl atoms. In the crystal structure, inter­molecular C—H⋯Cl hydrogen bonds link the mol­ecules into centrosymmetric dimers.

## Related literature

For related literature, see: Ahmadi, Kalateh *et al.* (2008 ▶); Ahmadi, Khalighi *et al.* (2008 ▶); Amani *et al.* (2007 ▶); Ilyukhin & Malyarick (1994 ▶); Khavasi *et al.* (2007 ▶); Khalighi *et al.* (2008 ▶); Malyarick *et al.* (1992 ▶); Nan *et al.* (1987 ▶); Yousefi, Khalighi *et al.* (2008 ▶); Yousefi, Tadayon Pour *et al.* (2008 ▶).
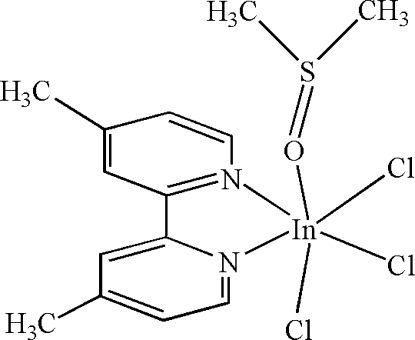


## Experimental

### 

#### Crystal data

[InCl_3_(C_12_H_12_N_2_)(C_2_H_6_OS)]
*M* *_r_* = 483.54
Monoclinic, 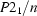

*a* = 8.2565 (17) Å
*b* = 23.456 (5) Å
*c* = 10.121 (2) Å
β = 105.95 (3)°
*V* = 1884.7 (7) Å^3^
*Z* = 4
Mo *K*α radiation
μ = 1.79 mm^−1^
*T* = 298 (2) K
0.49 × 0.46 × 0.44 mm

#### Data collection

Bruker SMART CCD area-detector diffractometer
Absorption correction: multi-scan (*SADABS*; Sheldrick, 1998 ▶) *T*_min_ = 0.404, *T*_max_ = 0.455
13791 measured reflections
5046 independent reflections
4804 reflections with *I* > 2σ(*I*)
*R*_int_ = 0.038

#### Refinement

*R*[*F* ^2^ > 2σ(*F* ^2^)] = 0.035
*wR*(*F* ^2^) = 0.085
*S* = 1.16
5046 reflections
201 parameters
H-atom parameters constrained
Δρ_max_ = 0.86 e Å^−3^
Δρ_min_ = −0.69 e Å^−3^

### 

Data collection: *SMART* (Bruker, 1998 ▶); cell refinement: *SAINT* (Bruker, 1998 ▶); data reduction: *SAINT*; program(s) used to solve structure: *SHELXTL* (Sheldrick, 2008 ▶); program(s) used to refine structure: *SHELXTL*; molecular graphics: *ORTEP-3 for Windows* (Farrugia, 1997 ▶) and *PLATON* (Spek, 2003 ▶); software used to prepare material for publication: *WinGX* (Farrugia, 1999 ▶).

## Supplementary Material

Crystal structure: contains datablocks I. DOI: 10.1107/S1600536808029553/hk2532sup1.cif

Structure factors: contains datablocks I. DOI: 10.1107/S1600536808029553/hk2532Isup2.hkl

Additional supplementary materials:  crystallographic information; 3D view; checkCIF report

## Figures and Tables

**Table:** 

Cl1—In1	2.4180 (12)
Cl2—In1	2.4592 (10)
Cl3—In1	2.4398 (9)
O1—In1	2.233 (2)
N1—In1	2.293 (2)
N2—In1	2.294 (2)

**Table:** 

Cl1—In1—Cl2	99.03 (4)
Cl1—In1—Cl3	98.15 (3)
Cl3—In1—Cl2	96.15 (3)
O1—In1—Cl1	90.56 (7)
O1—In1—Cl2	168.67 (6)
O1—In1—Cl3	88.39 (6)
O1—In1—N1	83.64 (9)
O1—In1—N2	79.94 (9)
N1—In1—Cl1	93.59 (7)
N1—In1—Cl2	89.72 (7)
N1—In1—Cl3	165.87 (6)
N2—In1—Cl1	162.86 (7)
N2—In1—Cl2	89.26 (7)
N2—In1—Cl3	95.83 (7)
N1—In1—N2	71.34 (9)

**Table 2:** Hydrogen-bond geometry (Å, °)

*D*—H⋯*A*	*D*—H	H⋯*A*	*D*⋯*A*	*D*—H⋯*A*
C1—H1⋯Cl1	0.93	2.77	3.427 (4)	128
C10—H10*C*⋯Cl2^i^	0.96	2.80	3.700 (4)	156

Symmetry code: (i) 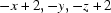.

**Fig. 1.**
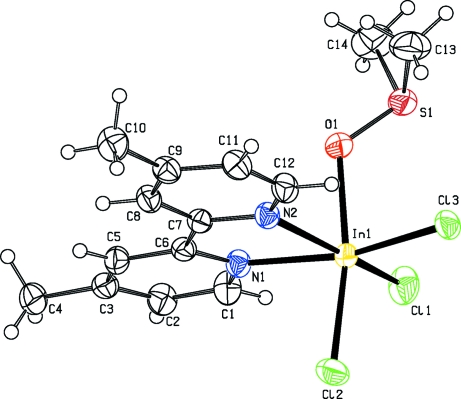
The molecular structure of the title molecule, with the atom-numbering scheme. Displacement ellipsoids are drawn at the 40% probability level.

**Fig. 2.**
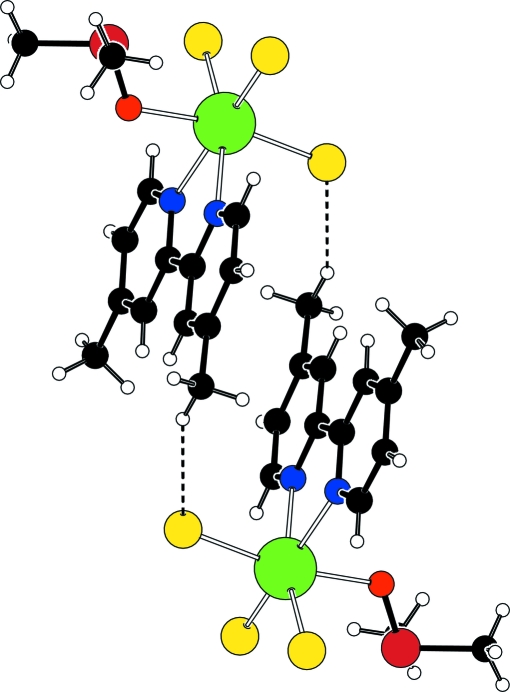
A partial packing diagram of the title compound. Hydrogen bonds are shown as dashed lines.

## supplementary crystallographic information

### Comment

Recently, we reported the syntheses and crystal structures of [Zn(5,5'-dmbpy)Cl_2_], (II), (Khalighi *et al.*, 2008), [Zn(6-mbpy)Cl_2_], (III), (Ahmadi, Kalateh *et al.*, 2008), [Cd(5,5'-dmbpy)(µ-Cl)_2_]_n_, (IV), (Ahmadi, Khalighi *et al.*, 2008), (Hg(4,4'-dmbpy)I_2_], (V), (Yousefi, Tadayon Pour *et al.*, 2008) and [Cu(5,5'-dcbpy)(en)(H_2_O)_2_].2.5H_2_O, (VI), (Yousefi, Khalighi *et al.*, 2008) [where 5,5'-dmbpy is 5,5'-dimethyl-2,2'-bipyridine, 6-mbpy is 6-methyl-2,2'-bipyridine, 4,4'-dmbpy is 4,4'-dimethyl-2,2'-bipyridine, 5,5'-dcbpy is 2,2'-bipyridine-5,5'-dicarboxylate and en is ethylenediamine]. We have also reported the synthesis and crystal structures of iron(III) complexes of [Fe(bipy)Cl_3_(DMSO)], (VII) and [Fe(phen)Cl_3_(DMSO)], (VIII), (Amani *et al.*, 2007) and [Fe(phen)Cl_3_(CH_3_OH)].CH_3_OH, (IX), (Khavasi *et al.*, 2007) [where bipy is 2,2'-bipyridine, DMSO is dimethyl sulfoxide and phen is 1,10-phenanthroline]. There are several In^III^ complexes, with formula, [In(N—N)Cl_3_(*L*)], (*L* = DMSO, H_2_O and EtOH), such as [In(bipy)Cl_3_(H_2_O)], (X), [In(bipy)Cl_3_(EtOH)], (XI) and [In(bipy)Cl_3_(H_2_O)].H_2_O, (XII), (Malyarick *et al.*, 1992), [In(phen)Cl_3_(DMSO)], (XIII), (Nan *et al.*, 1987), [In(phen)Cl_3_(H_2_O)], (XIV) and [In(phen)Cl_3_(EtOH)].EtOH, (XV), (Ilyukhin & Malyarick, 1994) have been synthesized and characterized by single-crystal X-ray diffraction methods. We report herein the synthesis and crystal structure of the title compound, (I).

In the title compound, (Fig. 1), the In^III^ atom is six-coordinated in a distorted octahedral configuration by two N atoms from 4,4'-dimethyl-2,2' -bipyridine, one O atom from  dimethyl sulfoxide and three Cl atoms. The In—Cl and In—N bond lengths and angles (Table 1) are within normal ranges, as in (XI), (XII), (XIII) and (XV).

In the crystal structure, intermolecular C-H···O hydrogen bonds (Table 2) link the molecules into centrosymmetric dimers (Fig. 2), in which they may be effective in the stabilization of the structure.

### Experimental

For the preparation of the title compound, (I), a solution of 4,4'-dimethyl -2,2'-bipyridine (0.20 g, 1.10 mmol) in methanol (10 ml) was added to a solution of InCl_3_.4H_2_O (0.16 g, 0.55 mmol) in methanol (5 ml) at room temperature. The suitable crystals for X-ray analysis were isolated after one week by methanol diffusion to a colorless solution in DMSO (yield; 0.19 g, 71.4%).

### Refinement

H atoms were positioned geometrically, with C-H = 0.93 and 0.96 Å for aromatic and methyl H, respectively, and constrained to ride on their parent atoms with U_iso_(H) = 1.2U_eq_(C).

### Crystal data

**Table:**

[InCl_3_(C_12_H_12_N_2_)(C_2_H_6_OS)]	*F*(000) = 960
*M**_r_* = 483.54	*D*_x_ = 1.704 Mg m^−^^3^
Monoclinic, *P*2_1_/*n*	Mo *K*α radiation, λ = 0.71073 Å
Hall symbol: -P 2yn	Cell parameters from 2052 reflections
*a* = 8.2565 (17) Å	θ = 1.7–29.3°
*b* = 23.456 (5) Å	µ = 1.79 mm^−^^1^
*c* = 10.121 (2) Å	*T* = 298 K
β = 105.95 (3)°	Prism, colorless
*V* = 1884.7 (7) Å^3^	0.49 × 0.46 × 0.44 mm
*Z* = 4	

### Data collection

**Table:**

Bruker SMART CCD area-detector diffractometer	5046 independent reflections
Radiation source: fine-focus sealed tube	4804 reflections with *I* > 2σ(*I*)
graphite	*R*_int_ = 0.038
φ and ω scans	θ_max_ = 29.3°, θ_min_ = 1.7°
Absorption correction: multi-scan (SADABS; Sheldrick, 1998)	*h* = −11→11
*T*_min_ = 0.404, *T*_max_ = 0.455	*k* = −28→32
13791 measured reflections	*l* = −13→12

### Refinement

**Table:**

Refinement on *F*^2^	Primary atom site location: structure-invariant direct methods
Least-squares matrix: full	Secondary atom site location: difference Fourier map
*R*[*F*^2^ > 2σ(*F*^2^)] = 0.036	Hydrogen site location: inferred from neighbouring sites
*wR*(*F*^2^) = 0.085	H-atom parameters constrained
*S* = 1.16	*w* = 1/[σ^2^(*F*_o_^2^) + (0.0263*P*)^2^ + 2.6542*P*] where *P* = (*F*_o_^2^ + 2*F*_c_^2^)/3
5046 reflections	(Δ/σ)_max_ = 0.018
201 parameters	Δρ_max_ = 0.86 e Å^−^^3^
0 restraints	Δρ_min_ = −0.69 e Å^−^^3^

### Special details

**Table:**

Geometry. All e.s.d.'s (except the e.s.d. in the dihedral angle between two l.s. planes) are estimated using the full covariance matrix. The cell e.s.d.'s are taken into account individually in the estimation of e.s.d.'s in distances, angles and torsion angles; correlations between e.s.d.'s in cell parameters are only used when they are defined by crystal symmetry. An approximate (isotropic) treatment of cell e.s.d.'s is used for estimating e.s.d.'s involving l.s. planes.
Refinement. Refinement of *F*^2^ against ALL reflections. The weighted *R*-factor *wR* and goodness of fit *S* are based on *F*^2^, conventional *R*-factors *R* are based on *F*, with *F* set to zero for negative *F*^2^. The threshold expression of *F*^2^ > σ(*F*^2^) is used only for calculating *R*-factors(gt) *etc*. and is not relevant to the choice of reflections for refinement. *R*-factors based on *F*^2^ are statistically about twice as large as those based on *F*, and *R*- factors based on ALL data will be even larger.

### Fractional atomic coordinates and isotropic or equivalent isotropic displacement parameters (Å^2^)

**Table:**

	*x*	*y*	*z*	*U*_iso_*/*U*_eq_	
In1	0.67799 (2)	0.143868 (8)	0.722848 (19)	0.03114 (7)	
Cl1	0.46060 (14)	0.15508 (4)	0.50881 (9)	0.0586 (2)	
Cl2	0.89844 (14)	0.10102 (4)	0.63344 (12)	0.0620 (3)	
Cl3	0.78999 (10)	0.24070 (3)	0.74202 (8)	0.04094 (16)	
S1	0.41807 (11)	0.23105 (4)	0.82159 (9)	0.04457 (18)	
O1	0.4998 (3)	0.17216 (10)	0.8405 (2)	0.0410 (5)	
N1	0.5893 (3)	0.05448 (10)	0.7626 (2)	0.0345 (5)	
N2	0.8245 (3)	0.11591 (10)	0.9400 (2)	0.0329 (5)	
C1	0.4701 (4)	0.02601 (15)	0.6694 (3)	0.0443 (7)	
H1	0.4125	0.0447	0.5892	0.053*	
C2	0.4290 (4)	−0.03006 (14)	0.6877 (3)	0.0440 (7)	
H2	0.3452	−0.0484	0.6207	0.053*	
C3	0.5131 (4)	−0.05872 (13)	0.8059 (3)	0.0378 (6)	
C4	0.4753 (5)	−0.12005 (14)	0.8289 (4)	0.0501 (8)	
H4A	0.4398	−0.1232	0.9113	0.060*	
H4B	0.5747	−0.1426	0.8378	0.060*	
H4C	0.3872	−0.1335	0.7522	0.060*	
C5	0.6370 (4)	−0.02882 (13)	0.9026 (3)	0.0369 (6)	
H5	0.6968	−0.0468	0.9832	0.044*	
C6	0.6714 (3)	0.02770 (12)	0.8790 (3)	0.0308 (5)	
C7	0.8006 (3)	0.06232 (12)	0.9795 (3)	0.0311 (5)	
C8	0.8859 (4)	0.04184 (13)	1.1078 (3)	0.0359 (6)	
H8	0.8674	0.0047	1.1325	0.043*	
C9	1.0000 (4)	0.07688 (13)	1.2006 (3)	0.0374 (6)	
C10	1.0914 (5)	0.05639 (17)	1.3414 (4)	0.0530 (9)	
H10A	1.0620	0.0799	1.4089	0.064*	
H10B	1.2106	0.0585	1.3532	0.064*	
H10C	1.0601	0.0176	1.3524	0.064*	
C11	1.0221 (4)	0.13209 (14)	1.1568 (3)	0.0403 (6)	
H11	1.0970	0.1569	1.2147	0.048*	
C12	0.9325 (4)	0.14978 (13)	1.0270 (3)	0.0400 (6)	
H12	0.9482	0.1867	0.9995	0.048*	
C13	0.5259 (7)	0.2699 (2)	0.9695 (5)	0.0731 (13)	
H13A	0.6407	0.2757	0.9686	0.088*	
H13B	0.5234	0.2489	1.0504	0.088*	
H13C	0.4720	0.3061	0.9699	0.088*	
C14	0.2249 (5)	0.2199 (2)	0.8626 (5)	0.0722 (13)	
H14A	0.2464	0.2014	0.9503	0.087*	
H14B	0.1527	0.1963	0.7935	0.087*	
H14C	0.1711	0.2559	0.8661	0.087*	

### Atomic displacement parameters (Å^2^)

**Table:**

	*U*^11^	*U*^22^	*U*^33^	*U*^12^	*U*^13^	*U*^23^
In1	0.03794 (11)	0.02645 (10)	0.03083 (10)	−0.00110 (7)	0.01249 (7)	0.00088 (7)
Cl1	0.0729 (6)	0.0538 (5)	0.0380 (4)	−0.0016 (4)	−0.0033 (4)	0.0062 (3)
Cl2	0.0772 (6)	0.0409 (4)	0.0877 (7)	0.0027 (4)	0.0561 (6)	−0.0066 (4)
Cl3	0.0448 (4)	0.0282 (3)	0.0536 (4)	−0.0030 (3)	0.0197 (3)	0.0006 (3)
S1	0.0475 (4)	0.0469 (4)	0.0438 (4)	0.0125 (3)	0.0200 (3)	0.0068 (3)
O1	0.0441 (11)	0.0412 (12)	0.0434 (11)	0.0053 (9)	0.0216 (9)	0.0042 (9)
N1	0.0379 (12)	0.0297 (12)	0.0350 (11)	−0.0049 (9)	0.0085 (9)	−0.0006 (9)
N2	0.0326 (11)	0.0296 (11)	0.0373 (12)	−0.0019 (9)	0.0111 (9)	0.0009 (9)
C1	0.0473 (17)	0.0394 (16)	0.0392 (15)	−0.0067 (14)	0.0002 (13)	0.0022 (12)
C2	0.0460 (17)	0.0384 (16)	0.0441 (16)	−0.0122 (13)	0.0067 (13)	−0.0062 (13)
C3	0.0455 (15)	0.0313 (14)	0.0406 (14)	−0.0055 (12)	0.0183 (12)	−0.0046 (11)
C4	0.068 (2)	0.0322 (16)	0.0527 (19)	−0.0109 (15)	0.0213 (17)	−0.0040 (14)
C5	0.0446 (15)	0.0312 (14)	0.0354 (13)	−0.0030 (12)	0.0116 (12)	0.0015 (11)
C6	0.0335 (12)	0.0290 (13)	0.0311 (12)	−0.0019 (10)	0.0109 (10)	−0.0028 (10)
C7	0.0329 (12)	0.0294 (13)	0.0321 (12)	0.0000 (10)	0.0109 (10)	−0.0018 (10)
C8	0.0405 (14)	0.0284 (13)	0.0382 (14)	−0.0002 (11)	0.0095 (11)	−0.0007 (11)
C9	0.0377 (14)	0.0364 (15)	0.0356 (14)	0.0029 (12)	0.0059 (11)	−0.0030 (11)
C10	0.061 (2)	0.051 (2)	0.0391 (16)	0.0022 (17)	−0.0007 (15)	0.0011 (14)
C11	0.0388 (15)	0.0365 (15)	0.0424 (15)	−0.0051 (12)	0.0058 (12)	−0.0067 (12)
C12	0.0416 (15)	0.0314 (14)	0.0478 (16)	−0.0073 (12)	0.0136 (13)	−0.0020 (12)
C13	0.091 (3)	0.064 (3)	0.079 (3)	−0.017 (2)	0.048 (3)	−0.028 (2)
C14	0.042 (2)	0.100 (4)	0.079 (3)	0.017 (2)	0.025 (2)	0.007 (3)

### Geometric parameters (Å, °)

**Table:**

Cl1—In1	2.4180 (12)	C7—N2	1.350 (4)
Cl2—In1	2.4592 (10)	C7—C8	1.382 (4)
Cl3—In1	2.4398 (9)	C8—C9	1.400 (4)
O1—In1	2.233 (2)	C8—H8	0.9300
O1—S1	1.526 (2)	C9—C11	1.397 (4)
N1—In1	2.293 (2)	C9—C10	1.497 (4)
N2—In1	2.294 (2)	C10—H10A	0.9600
C1—N1	1.339 (4)	C10—H10B	0.9600
C1—C2	1.383 (5)	C10—H10C	0.9600
C1—H1	0.9300	C11—C12	1.382 (5)
C2—C3	1.382 (5)	C11—H11	0.9300
C2—H2	0.9300	C12—N2	1.331 (4)
C3—C5	1.395 (4)	C12—H12	0.9300
C3—C4	1.504 (4)	C13—S1	1.770 (5)
C4—H4A	0.9600	C13—H13A	0.9600
C4—H4B	0.9600	C13—H13B	0.9600
C4—H4C	0.9600	C13—H13C	0.9600
C5—C6	1.390 (4)	C14—S1	1.775 (4)
C5—H5	0.9300	C14—H14A	0.9600
C6—N1	1.343 (4)	C14—H14B	0.9600
C6—C7	1.495 (4)	C14—H14C	0.9600
			
Cl1—In1—Cl2	99.03 (4)	H4B—C4—H4C	109.5
Cl1—In1—Cl3	98.15 (3)	C6—C5—C3	120.3 (3)
Cl3—In1—Cl2	96.15 (3)	C6—C5—H5	119.9
O1—In1—Cl1	90.56 (7)	C3—C5—H5	119.9
O1—In1—Cl2	168.67 (6)	N1—C6—C5	121.3 (3)
O1—In1—Cl3	88.39 (6)	N1—C6—C7	115.9 (2)
O1—In1—N1	83.64 (9)	C5—C6—C7	122.8 (3)
O1—In1—N2	79.94 (9)	N2—C7—C8	121.6 (3)
N1—In1—Cl1	93.59 (7)	N2—C7—C6	116.1 (2)
N1—In1—Cl2	89.72 (7)	C8—C7—C6	122.3 (3)
N1—In1—Cl3	165.87 (6)	C7—C8—C9	120.1 (3)
N2—In1—Cl1	162.86 (7)	C7—C8—H8	119.9
N2—In1—Cl2	89.26 (7)	C9—C8—H8	119.9
N2—In1—Cl3	95.83 (7)	C11—C9—C8	117.0 (3)
N1—In1—N2	71.34 (9)	C11—C9—C10	121.6 (3)
O1—S1—C13	104.9 (2)	C8—C9—C10	121.4 (3)
O1—S1—C14	103.4 (2)	C9—C10—H10A	109.5
C13—S1—C14	98.8 (2)	C9—C10—H10B	109.5
H14B—C14—H14C	109.5	H10A—C10—H10B	109.5
S1—O1—In1	122.38 (12)	C9—C10—H10C	109.5
C1—N1—C6	118.7 (3)	H10A—C10—H10C	109.5
C1—N1—In1	122.7 (2)	H10B—C10—H10C	109.5
C6—N1—In1	118.26 (18)	C12—C11—C9	119.8 (3)
C12—N2—C7	119.1 (3)	C12—C11—H11	120.1
C12—N2—In1	123.0 (2)	C9—C11—H11	120.1
C7—N2—In1	117.94 (18)	N2—C12—C11	122.4 (3)
N1—C1—C2	122.7 (3)	N2—C12—H12	118.8
N1—C1—H1	118.7	C11—C12—H12	118.8
C2—C1—H1	118.7	S1—C13—H13A	109.5
C3—C2—C1	119.7 (3)	S1—C13—H13B	109.5
C3—C2—H2	120.2	H13A—C13—H13B	109.5
C1—C2—H2	120.2	S1—C13—H13C	109.5
C2—C3—C5	117.4 (3)	H13A—C13—H13C	109.5
C2—C3—C4	121.6 (3)	H13B—C13—H13C	109.5
C5—C3—C4	121.1 (3)	S1—C14—H14A	109.5
C3—C4—H4A	109.5	S1—C14—H14B	109.5
C3—C4—H4B	109.5	H14A—C14—H14B	109.5
H4A—C4—H4B	109.5	S1—C14—H14C	109.5
C3—C4—H4C	109.5	H14A—C14—H14C	109.5
H4A—C4—H4C	109.5		
			
S1—O1—In1—N1	−154.51 (17)	N1—C1—C2—C3	−0.1 (5)
S1—O1—In1—N2	133.38 (17)	C1—C2—C3—C5	0.2 (5)
S1—O1—In1—Cl1	−60.97 (15)	C1—C2—C3—C4	−178.7 (3)
S1—O1—In1—Cl3	37.17 (15)	C2—C3—C5—C6	0.4 (5)
S1—O1—In1—Cl2	151.0 (2)	C4—C3—C5—C6	179.4 (3)
C1—N1—In1—O1	98.2 (3)	C3—C5—C6—N1	−1.2 (4)
C6—N1—In1—O1	−88.1 (2)	C3—C5—C6—C7	179.0 (3)
C1—N1—In1—N2	179.7 (3)	C5—C6—N1—C1	1.3 (4)
C6—N1—In1—N2	−6.6 (2)	C7—C6—N1—C1	−178.9 (3)
C1—N1—In1—Cl1	8.0 (3)	C5—C6—N1—In1	−172.7 (2)
C6—N1—In1—Cl1	−178.3 (2)	C7—C6—N1—In1	7.1 (3)
C1—N1—In1—Cl3	154.2 (2)	N1—C6—C7—N2	−2.2 (4)
C6—N1—In1—Cl3	−32.1 (4)	C5—C6—C7—N2	177.6 (3)
C1—N1—In1—Cl2	−91.0 (3)	N1—C6—C7—C8	175.1 (3)
C6—N1—In1—Cl2	82.7 (2)	C5—C6—C7—C8	−5.1 (4)
C12—N2—In1—O1	−88.9 (2)	C8—C7—N2—C12	−0.2 (4)
C7—N2—In1—O1	91.9 (2)	C6—C7—N2—C12	177.1 (3)
C12—N2—In1—N1	−175.5 (3)	C8—C7—N2—In1	179.0 (2)
C7—N2—In1—N1	5.30 (19)	C6—C7—N2—In1	−3.7 (3)
C12—N2—In1—Cl1	−146.1 (2)	N2—C7—C8—C9	0.2 (4)
C7—N2—In1—Cl1	34.7 (4)	C6—C7—C8—C9	−177.0 (3)
C12—N2—In1—Cl3	−1.6 (2)	C7—C8—C9—C11	−0.2 (4)
C7—N2—In1—Cl3	179.24 (19)	C7—C8—C9—C10	178.6 (3)
C12—N2—In1—Cl2	94.5 (2)	C8—C9—C11—C12	0.3 (5)
C7—N2—In1—Cl2	−84.65 (19)	C10—C9—C11—C12	−178.5 (3)
In1—O1—S1—C13	−105.2 (2)	C9—C11—C12—N2	−0.3 (5)
In1—O1—S1—C14	151.6 (2)	C11—C12—N2—C7	0.3 (5)
C2—C1—N1—C6	−0.6 (5)	C11—C12—N2—In1	−178.9 (2)
C2—C1—N1—In1	173.1 (3)		

### Hydrogen-bond geometry (Å, °)

**Table:**

*D*—H···*A*	*D*—H	H···*A*	*D*···*A*	*D*—H···*A*
C1—H1···Cl1	0.93	2.77	3.427 (4)	128.
C10—H10C···Cl2^i^	0.96	2.80	3.700 (4)	156.

Symmetry codes: (i) −*x*+2, −*y*, −*z*+2.
